# Growing in time: exploring the molecular mechanisms of tree growth

**DOI:** 10.1093/treephys/tpaa065

**Published:** 2020-05-29

**Authors:** Rajesh Kumar Singh, Rishikesh P Bhalerao, Maria E Eriksson

**Affiliations:** 1 Department of Plant Physiology, Umeå Plant Science Centre, Umeå University, Umeå SE-901 87, Sweden; 2 Department of Forest Genetics and Plant Physiology, Umeå Plant Science Centre, Swedish University of Agricultural Sciences, Umeå SE-901 82, Sweden

**Keywords:** cell cycle, circadian clock, cytokinins, gibberellins, growth, phenology

## Abstract

Trees cover vast areas of the Earth’s landmasses. They mitigate erosion, capture carbon dioxide, produce oxygen and support biodiversity, and also are a source of food, raw materials and energy for human populations. Understanding the growth cycles of trees is fundamental for many areas of research. Trees, like most other organisms, have evolved a circadian clock to synchronize their growth and development with the daily and seasonal cycles of the environment. These regular changes in light, daylength and temperature are perceived via a range of dedicated receptors and cause resetting of the circadian clock to local time. This allows anticipation of daily and seasonal fluctuations and enables trees to co-ordinate their metabolism and physiology to ensure vital processes occur at the optimal times. In this review, we explore the current state of knowledge concerning the regulation of growth and seasonal dormancy in trees, using information drawn from model systems such as *Populus* spp.

## Introduction

The combination of the Earth’s orbit with its daily rotation about a tilted axis produces regular changes in daylength, which lead to changes in temperature, humidity and often precipitation. As a result, many organisms, including plants, have evolved an inner timing mechanism that resonates with the external 24-hour day/ night cycle.

Scientists have long been interested in rhythmic phenomena in plants, with Jean Jacques d’Ortous de Mairan, Carl von Linné and Charles and Francis Darwin being notable early experimentalists in this field (De Mairan 1729, Linné 1755, Darwin and Darwin 1881, Sweeney 1969). The coupling between inner timing and the external environment was conceptualized in the mid-20th century when chronobiologists proposed models of the innate timekeeping mechanism, as well as theories to account for its interactions with light and photoperiod (Garner and Allard 1922, Bünning 1936, Pittendrigh and Minis 1964). Later, genetic and molecular experimentation revealed that an internal oscillator (‘clock’) was responsible for timekeeping in most organisms (Young and Kay 2001, Rosbash 2009). This oscillator has evolved to run with an innate circadian period of about 24 hours. It allows the organism to reset to local time and to anticipate regular environmental changes (McWatters and Devlin 2011). Under natural conditions, circadian oscillators are synchronized or reset by zeitgebers (‘time-givers’) such as the regular changes in light and temperature (McWatters and Devlin 2011, Millar 2016, McClung 2019). A properly functioning clock may increase growth, reproductive success, competitiveness and survival (Ouyang et al. 1998, Green et al. 2002, Michael et al. 2003, Dodd et al. 2007, O'Donnell et al. 2011, Rubin et al. 2017).

As plants are sessile and cannot move to avoid hostile conditions or pathogen attack, it is crucial that their internal metabolism matches their external environment. Plant rhythms include daily movements of leaves and flowers, as well as seasonal events such as flowering and bud set (Thomas and Vince Prue 1997, Millar 2016). Although daylength (photoperiod) variation and seasonal cyclicity are most pronounced at higher latitudes (Thomas and Vince Prue 1997, Cooke et al. 2012), photoperiod is also a dominant factor regulating growth at lower latitudes (Adole et al. 2019). In temperate climates, periods of growth of perennial species alternate with times of growth arrest and dormancy in response to growth-inhibiting short days (SDs) (Thomas and Vince Prue 1997, Cooke et al. 2012). Growth cessation and bud set occur at the apical meristems of deciduous species (Rohde et al. 2002, Rohde and Bhalerao 2007) and are primarily controlled by daylength shifts that induce major remodelling of the transcriptome (Ruttink et al. 2007, Hoffman et al. 2010, Karlberg et al. 2010, Filichkin et al. 2011).

Photoperiodic control of growth in trees relies on the circadian clock (Thomas and Vince Prue 1997, Lagercrantz 2009, Ibáñez et al. 2010, Kozarewa et al. 2010, Gyllenstrand et al. 2014). Senescence and leaf fall are associated with daylength shortening in autumn and rely on SDs, low temperature and metabolic cues (Lagercrantz 2009, Michelson et al. 2018). Accurate timing of growth arrest also confers resistance to drought and/or freezing. In spring, when days become warmer and longer, the start of the growing season is marked by bud flushing; a timely bud flush relies on a functional circadian clock, in some tree species at least (Thomas and Vince Prue 1997, Lagercrantz 2009, Cooke et al. 2012). The timely completion of these crucial processes is a prerequisite for the survival of woody species at high latitudes.

Regulation of growth and flowering in plants relies on interactions between the clock and light. Light, received by photoreceptors, resets the phase and pace of the clock to align it with the environment. The ‘external coincidence’ model of photoperiodism (Bünning 1960, Pittendrigh 1972) postulates that the clock controls rhythms of gene and protein expression in order to define a particular part of the cycle as the light-sensitive phase. Recent work in Arabidopsis (*Arabidopsis thaliana*) and *Populus* spp. indicates that external coincidence between the light/dark cycle and rhythms of protein expression underlies control of flowering and growth (Putterill et al. 1995, Thomas and Vince Prue 1997, Piñeiro and Coupland 1998, Fowler et al. 1999, Carre 2001, Suarez-Lopez et al. 2001, Valverde et al. 2004, Böhlenius et al. 2006, Imaizumi and Kay 2006, Corbesier et al. 2007, Lagercrantz 2009, Kozarewa et al. 2010, Cooke et al. 2012, Ding et al. 2018, Ramos-Sánchez et al. 2019).

The circadian clock plays a fundamental role in both herbaceous annuals and biennials, and perennial species. Studies of angiosperms, including *Populus* spp*., Castanea sativa* and *Eucalyptus* spp., and of gymnosperms such as *Picea abies*, have elucidated many aspects of the daily and seasonal control of growth (Rhode et al. 1999, Allona et al. 2008, Lagercrantz 2009, Cooke et al. 2012, Johansson et al. 2015, Ding and Nilsson 2016). This review focusses on the regulatory mechanisms that depend on the proper processing of light and temperature cues by the circadian clock to control plant development and coordinate the seasonal cycles of vegetative growth and dormancy.

## Environmental factors controlling plant growth

### Light and temperature perception and regulation

**Figure 1.**
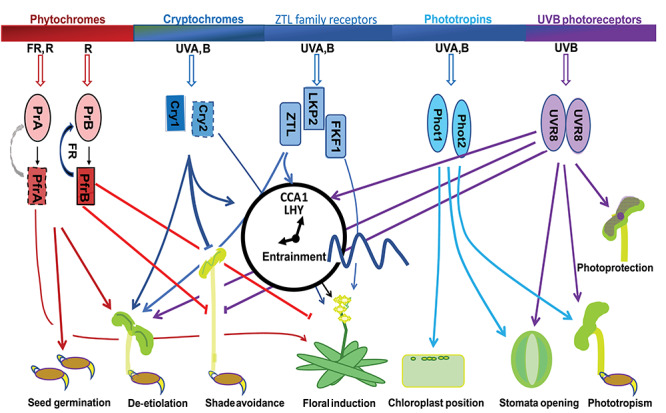
A schematic outline of plant photoreception across the light spectrum. Coloured lines indicate the contributions of each photoreceptor to plant development and responses. Activated phytochrome A (PfrA) and cryptochrome 2 (cry2) are highly unstable (indicated by dashed outlines or grey arrow), unlike other activated phytochromes or cryptochrome 1 (cry1). Arrows suggest a promotive interaction while bars indicate a repression of the activity.

A diverse set of photoreceptors have evolved in plants to transduce information about light quality, irradiance, direction and duration to control growth and development, and their roles have been most elucidated in Arabidopsis (Figure 1). The most important of these photoreceptors respond to blue, red and far-red wavelengths. Although these wavelengths provide energy for photosynthesis, they also act as key signals for plant development and growth (Zhen and van Iersel 2017). Phytochromes (phys) are photoreceptors that absorb red (660 nm), far-red (730 nm) and blue light (470 nm). There are five functional *PHY* genes (*PHYA–E*) in the Arabidopsis genome (Sharrock and Quail 1989, Clack et al. 1994, Quail et al. 1994, Neff and Chory 1998, Whitelam and Devlin 1998, Franklin and Quail 2010, Galvão and Fankhauser 2015). Each *PHY* gene encodes an apoprotein (Quail et al. 1994) that is post-translationally converted to the holoprotein (phy) by the covalent attachment of a linear tetrapyrrole pigment. Phy holoproteins act in partnership as homo- or heterodimers. When excited by red (or blue) light, the phy protein goes through a *cis*-to-*trans* photoisomerization in the C15–C16 double bond between the C and D rings of the linear tetrapyrrole. This transition converts inactive Pr to biologically active Pfr (Li et al. 2011). Phys in the Pfr state act in the cytosol to open cytosolic calcium channels, causing the release of cGMP, which activates transcription factors (Li et al. 2011). The physical transformation to the Pfr form results in the translocation of the dimer from the cytosol to the nucleus, where it acts directly in transcription factor complexes to control gene expression in response to light (Ni et al. 1998, Tepperman et al. 2001, Franklin et al. 2011, Li et al. 2011). A pulse of far-red light prompts a conformational change in light-stable phys (phyB–E) from Pfr to Pr, but causes ubiquitination and degradation of the light-labile phyA (Seo et al. 2004, Franklin and Quail 2010, Li et al. 2011).

The phys are only a part, however, of a diverse array of plant photoreceptors that rely on attached chromophores to perceive light. For photoreceptors sensitive to blue and ultraviolet-A (UVA) light, this is typically a prosthetic flavin group (flavin mononucleotide—FMN), flavin adenine dinucleotide (FAD) or 5,10-methenyltetrahydrofolate (MTHF) attached to the apoprotein. The prosthetic group activates the protein following excitation by light of the appropriate wavelength (Christie et al. 2015). The cryptochrome (cry) holoproteins contain both FAD and MTHF chromophores. Cry1 and cry2 are blue light receptors that mainly respond to blue (400–500 nm) or UVA (315–400 nm) light, but are also somewhat sensitive to green light (520–560 nm) (Liu et al. 2011). The Arabidopsis genome also encodes a third cry (*CRY3*) whose action resembles a photolyase (Liu et al. 2011, Christie et al. 2015). Together, the phys and crys control major events in plant development, including germination, de-etiolation and flowering (Lin and Todo 2005, Galvão and Fankhauser 2015). They are expressed rhythmically and, in *Populus* spp*.*, appear to be targets of LATE ELONGATED HYPOCOTYL 1 (LHY1) and/or LHY2, as their expression shifts in trees with reduced levels of *LHY1* and *LHY2* gene expression (Edwards et al. 2018).

Other photoreceptors that respond to blue and UVA light are members of the ZEITLUPE/ADAGIO (ZTL/ADO1) family and the phototropins (phots) flavoproteins. These all contain oxidized FMN as a prosthetic group at their specific light, oxygen or voltage sensing (LOV) domains (Christie et al. 1999, Mizoguchi and Coupland 2000, Nelson et al. 2000, Somers et al. 2000, Jarillo et al. 2001, Schultz et al. 2001, Kim et al. 2007). The main roles of ZTL family proteins to regulate the speed of the circadian oscillator (ZTL) and sense photoperiod (FLAVIN-BINDING, KELCH REPEAT, F BOX 1 (FKF1); LOV KELCH PROTEIN 2 (LKP2)), but they are also involved in de-etiolation processes such as the inhibition of hypocotyl elongation (Somers et al. 2000, Jarillo et al. 2001). All ZTL family members share significant homology. FKF1 and LKP2 have complementary roles to ZTL and act in its absence to control the speed of the oscillator (Baudry et al. 2010). In *Populus,* FKF1 acts in photoperiodic sensing (Ding et al. 2018) whilst ZTL controls the speed of the circadian oscillator as well as growth cessation and bud set (Eriksson ME, Ibáñez C, Kozarewa I unpublished).

Phot1 and phot2 control phototropism, stomatal opening and chloroplast movements in Arabidopsis (Yin and Ulm 2017). In *Populus*, several gene models corresponding to *PHOT1* and *PHOT2* are rhythmically expressed and affected by LHY1 and/or LHY2 expression (Edwards et al. 2018), and at least five tentative gene models are targets of LHY2 (Ramos-Sánchez et al. 2019).

UV RESISTANCE LOCUS 8 (UVR8) is a photoreceptor capable of detecting the more energetic UVB (280–315 nm) wavelengths (Rizzini et al. 2011). The protein forms dimers which detect UVB. UVR8 influences light-regulated plant development in similar and complementary ways to other photoreceptors, being involved in de-etiolation, UVB light acclimation and tolerance, inhibition of phototropism, inhibition of shade avoidance responses, leaf development and stomatal regulation (Tilbrook et al. 2013, Yin and Ulm 2017). It also acts in DNA repair, in mitigating oxidative stress and in photoinhibition (Tilbrook et al. 2013, Yin and Ulm 2017). *UVR8, REPRESSOR OF UV-B PHOTOMORPHOGENESIS 2 (RUP2)* and other factors associated with UV-responses are co-expressed with *LHY1* and *LHY2*, and their expression shifts in trees with reduced levels of *LHY1* and *LHY2* (Edwards et al. 2018). Although only the phys mediate germination, all the photoreceptors other than the phots are involved in seedling de-etiolation and in resetting the circadian oscillator to the local day/night cycle (Millar 2003). The phots may modulate circadian rhythms in operating efficiency of the photosystem II (Litthauer et al. 2015). Importantly, the circadian clock also acts to control or ‘gate’ sensitivity to photoreceptor action (Millar 2003). In Arabidopsis, the phots and UVR8 regulate stomatal opening and phototropism, as well as chloroplast movement and photoprotection (Tilbrook et al. 2013, Yin and Ulm 2017) (Figure 1). Although many of the functions ascribed to photoreceptors in Arabidopsis appear similar in trees, further experimentation is required to provide a detailed understanding of the different systems.

Light has a major effect on plant growth. Environmental light signals are detected by dedicated photoreceptors (predominantly the phys), integrated by the circadian clock, and then fed into the pathways regulating photomorphogenesis (Ni et al. 1998, Tepperman et al. 2001, McWatters and Devlin 2011, Gangappa and Botto 2016, Hajdu et al. 2018). The PHYTOCHROME INTERACTING FACTORS (PIF1, PIF3, PIF4, PIF5, PIF6, PIF7 and PIF8) are a family of HELIX-LOOP-HELIX type transcription factors that are negative regulators of photomorphogenesis and growth (Toledo-Ortiz et al. 2003). In contrast, ELONGATED HYPOCOTYL 5 (HY5), together with transcription factors such as LONG HYPOCOTYL IN FAR-RED (HFR1), promotes photomorphogenesis (Gangappa and Botto 2016). The interplay between these different pathways determines seedling development, as well as a plant’s responses to the daily fluctuations in light quality. Detection of light by phys causes destabilization of PIFs, which are then degraded via the 26s proteasome (Xu et al. 2017). The circadian PSEUDORESPONSE REGULATORS (PRRs) are directly involved in controlling the expression and action of PIFs. PRR9, PRR7, PRR5 and PRR1/TIMING OF CAB EXPRESSION 1 (TOC1) are expressed in waves between dawn and dusk and, by a combination of tightly controlled transcription and translation with direct binding to PIF proteins, inhibit the ability of PIFs to bind to shared *cis*-elements, thus gating responses such as hypocotyl elongation (Strayer et al. 2000, Eriksson et al. 2003, Kaczorowski and Quail 2003, Nakamichi et al. 2005, Leivar et al. 2008, Soy et al. 2016, Zhu et al. 2016, Martín et al. 2018). The control of flowering time in Arabidopsis requires the PRR quintet to take turns in restricting the ability of CYCLING DOF FACTORS (CDFs) to inhibit *CONSTANS (CO)* expression as well as stabilizing the CO protein through direct interactions (Nakamichi et al. 2007, 2012, Fornara et al. 2009, Hayama et al. 2017). The ability of clock components to act downstream of photoreceptors to restrict gene transcription and protein levels to specific points in the 24-hour cycle (‘gating’), thus periodically inhibiting binding of core transcription factors, appears to be a general regulatory mechanism for the efficient control of development and growth, across the day as well as the different seasons of the year.

Under natural conditions, light and temperature signals are intertwined and reinforce each other. These environmental cues regulate many developmental processes from germination to de-etiolation and flowering (Li et al. 2011). Temperature is perceived in several ways by plants (Penfield 2008) but the complex thermosensory mechanism was, until recently, poorly understood. It is now clear that a number of photoreceptors including phyB (Jung et al. 2016, Legris et al. 2016, Casal and Balasubramanian 2019), phot1 (Fujii et al. 2017), cry1 (Ma et al. 2016) and UVR8 (Hayes et al. 2017) respond to temperature and thus integrate light and temperature cues. Moreover, temperature shifts mediate changes at the level of the membrane (Martinière et al. 2011) and chromatin/chromatin-activity state (De Lucia et al. 2008, Kumar and Wigge 2010), enabling adaptation. The circadian clock integrates thermal information and regulates physiological sensitivity to such signals, thus buffering the plant against short-term changes, as well as controlling transcription of a very large number of genes (Gould et al. 2006, 2013).

### The circadian system in trees

**Figure 2.**
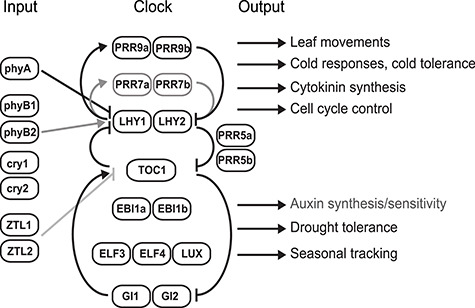
A simplified outline of the circadian system of *Populus* showing inputs, central clock (oscillator) and outputs. The clock ensures that growth and other processes are synchronized with the local environment, time of day and season. LHY1, LHY2 and TOC1 are core components of the oscillator whose functions are complemented by additional elements. Cell proliferation is controlled by the morning clock components LHY1 and LHY2 via cytokinin biosynthesis and CYCLIN D3; auxin is regulated in the evening. EBI1a and/or EBI1b are likely to act at night, restricting LHY1 and LHY2 expression. Arrows show positive effects; bars show negative effects. Black text and lines show pathways with experimental support; grey text and lines show tentative pathways.

The circadian system consists of inputs, clock (oscillator) and outputs. Detailed discussions of the Arabidopsis clock and its function can be found elsewhere (Nagel and Kay 2012, Fogelmark and Troein 2014). Although most Arabidopsis genes associated with the clock have orthologues in *Populus*, in several cases only one orthologue is found in the *Populus trichocarpa* genome despite the recent genome duplication event in this genus (Tuskan et al. 2006). Figure 2 shows an outline of the circadian system of *Populus* constructed from homologies with the Arabidopsis clock, (Ibáñez et al. 2010, Kozarewa et al. 2010, Edwards et al. 2018, Ramos-Sánchez et al. 2019, Takata et al. 2009, 2010, Eriksson et al. unpublished). The morning-phased transcription factors LHY1 and LHY2, together with the evening-phased protein TOC1, are important components of the central circadian oscillator of *Populus* (Kozarewa et al. 2010). In the morning, far-red light is detected by photoreceptors including phyA and transduced to LHY1 and LHY2. The functions of LHY1, LHY2 and TOC1 in the core oscillator are complemented by a number of additional components that modulate clock speed and responses to environmental cues (Figure 2).

The oscillator controls a large number of output processes, including leaf movements (Kozarewa et al. 2010), cold responses, freezing tolerance (Ibáñez et al. 2010), water use and stress (Resco de Dios et al. 2016, Ke et al. 2017) and seasonal tracking (Ding et al. 2018). The regulation of auxin levels (diurnal pattern) and sensitivity may also be under clock control (Harmer et al. 2000, Covington and Harmer 2007, Baba et al. 2011, Edwards et al. 2018). TOC1, LHY1 and LHY2 act together to control growth, wood formation and level of biomass production (Ibáñez et al. 2010, Edwards et al. 2018). LHY1 and LHY2 also control cell proliferation via cytokinin biosynthesis and CYCLIN D3 (CYCD3); the pattern of auxin expression suggests it is regulated by the evening clock (Edwards et al. 2018). In Arabidopsis, a nuclear transcription factor, EARLY BIRD (EBI), an X box-binding-like (NFXL) protein, alters the speed of the circadian clock (Ashelford et al. 2011, Mikael Johansson et al. 2011); in *Populus* spp., its orthologues EBI1a and EBI1b regulate clock function and growth (Eriksson et al. 2018). EBI1a and/or EBI1b are likely to act in the oscillator at night, perhaps by restricting LHY1 and LHY2 expression to the morning, thus affecting the timing of growth.

### Internal control

Dissection of the circadian oscillators in a variety of different organisms including cyanobacteria, fungi and animals has established a common theme but not a high conservation of genes and proteins. Circadian systems are conceptually similar across a wide range of phyla, being made up of an input signal pathway that entrains the oscillator, the oscillator itself that generates the periodicity, and a set of output pathways that determine an individual cell’s physiological state or an entire organism’s development, growth and activity (Figure 2). The oscillator also controls its own sensitivity to resetting cues, a phenomenon known as ‘gating’. Gating that renders an oscillator particularly perceptive to signals at dawn and dusk is called ‘parametric entrainment’. Other entrainment mechanisms are also possible; for example, ‘non-parametric entrainment’ was described by Jürgen Aschoff, who noted that the speed of the oscillator (period length) is inversely proportional to light intensity in day-active organisms (the opposite situation being observed in night-active organisms) (Aschoff 1960). Both types of entrainment occur under natural conditions to enable an organism to stay on local time.

Although changes in temperature can reset the oscillator, a shared feature of circadian oscillators is that they are temperature compensated. Temperature compensation allows the clock to function across a range of temperature without a large change in period; for Arabidopsis, this range is generally between 12 and 27 °C (Gould et al. 2006), as plants become arrhythmic at lower temperatures (Bieniawska et al. 2008, Dong et al. 2011, Chow et al. 2014). Thus, the circadian system is buffered against temperature fluctuations or changes in ambient temperature.

Temperature is an important factor in the lives of perennial species such as trees. Studies of the impact on phenology of changes in climate have shown that growth cessation and bud set are strongly affected by temperature in an ecotype-dependent manner (Cooke et al. 2012). In some species, however, photoperiod is the major environmental signal whereas in others, such as apple (*Malus domestica*) and other members of the Rosaceae, temperature is the major seasonal cue regulating phenology, with low temperatures inducing bud set and dormancy (Heide and Prestrud 2005). Temperate plant species increase their freezing tolerance in response to a prolonged period of low non-freezing temperatures, a process known as ‘cold acclimation’ (Thomashow 1999). Northern ecotypes tend to stop and resume growth early, while southern ecotypes are later to respond, possibly to avoid mistiming if short spells of warm weather occur early in spring; such spells pose a threat because forest trees are most vulnerable to death by freezing following premature growth.

Few studies have addressed molecular responses to temperature, and those that have focussed mostly on low temperature responses. In both *C. sativa* and *Populus* spp*.*, cold causes constant, high expression of central circadian clock components (Ramos et al. 2005, Ibáñez et al. 2008, 2010). In *Populus* spp*.*, LHY1 and LHY2 (orthologous to, respectively, CIRCADIAN CLOCK ASSOCIATED 1 (CCA1) and LHY in Arabidopsis) control expression of *C-REPEAT-BINDING FACTOR/DEHYDRATION RESPONSIVE ELEMENT-BINDING FACTOR 1* (*CBF/DREB1*)*,* as well as the cold response and freezing tolerance (Ibáñez et al. 2010). In Arabidopsis, the single MYB domain transcription factors CCA1 and LHY are clock components that directly activate the CBF signalling cascade and thus confer low temperature resilience (Bieniawska et al. 2008, Espinoza et al. 2010, Dong et al. 2011). In both Arabidopsis and *Populus* spp., proper regulation of the phy photoreceptors is required for normal circadian period and control of phase (Somers et al. 1998, Salomé et al. 2002, Kozarewa et al. 2010).

The finding that CCA1 and LHY control responses to cold and freezing matches the more recent observation that the amplitude of LHY oscillations increases when daylength is shorter than the critical daylength (CDL; the minimum daylength permissive of flowering or growth), implying that LHY may stop growth and boost levels of CBFs, enabling basal cold tolerance (Hoffman et al. 2010, Ibáñez et al. 2010, Ramos-Sánchez et al. 2019). At low temperatures, these signalling pathways increase the levels of expression of more extensive cold regulons; in some species this is enough to build up sufficient freezing tolerance to withstand exposure to liquid nitrogen (−196 °C) (Eriksson and Webb 2011). Ramos-Sánchez et al. (2019) showed that LHY2 binds to the promoters of many genes following exposure to photoperiods shorter than the CDL. The direct targets of LHY2 include *COLD-RESPONSIVE PROTEIN KINASE 1 (CRPK1)* and *MEDIATOR16/SENSITIVE TO FREEZING 6* (*MED16/SFR6*); both are likely essential for the proper transcription and post-translational regulation of CBF proteins in *Populus* as their orthologues regulate cold responses in Arabidopsis (Knight et al. 2008, Hemsley et al. 2014, Liu et al. 2017). CRPK1 in the plasma membrane is activated by cold stress and phosphorylates 14-3-3 proteins, which then translocate from the cytoplasm to the nucleus and promote the degradation of CBFs via the 26s proteasome (Liu et al. 2017). MED16/SFR6 acts in a mediator complex to promote expression of CBF targets and other cold-responsive genes (Knight et al. 2008, Hemsley et al. 2014). Both CRPK1 and SFR6 are thus likely to be required for freezing tolerance under SDs.

## Timing of growth in context

Many studies on organisms ranging from cyanobacteria to fungi and higher plants have suggested that individuals whose innate circadian period matches (resonates with) that of the external cycle grow and reproduce better than mismatched competitors (Ouyang et al. 1998, Dodd et al. 2005, Hevia et al. 2015). Cyanobacteria strains with such matched cycles outcompete unmatched competitors under a range of conditions (Ouyang et al. 1998). Arabidopsis ecotypes collected along a latitudinal cline show a correlation between circadian period and leaf angle, with ecotypes from high latitudes having longer periods. This suggests the circadian oscillator is adapted to optimize the position of the leaf for photosynthesis and to ensure flowering occurs later in the year to coincide with summer at those latitudes (Michael et al. 2003). Photosynthesis, biomass accumulation and fitness all increase in Arabidopsis plants whose circadian period matches the external environmental rhythms (Dodd et al. 2005). Again in Arabidopsis, starch accumulation and mobilization are under circadian control (Blasing et al. 2005, Graf et al. 2010) and metabolic sugars are able to reset the circadian oscillator in the morning (Haydon et al. 2013, Shin et al. 2017, Frank et al. 2018).

Selective breeding of crops such as barley (*Hordeum vulgare*), tomato (*Solanum lycopersicum*) and rice (*Oryza sativa*) has resulted in changes in photoreception as well as in the circadian clock, increasing productivity at higher latitudes. Studies of tomato varieties across the broad latitudinal cline between Chile/Peru and Mexico indicate that selective breeding has produced plants with a longer period (Müller et al. 2015, 2018), presumably for similar reasons to those described by Michael et al. (2003) for high latitude ecotypes of Arabidopsis. Thus, a functioning circadian clock with the correct relationship with the environmental pattern of light and dark ensures successful timing of growth cessation, dormancy and freezing tolerance. Early studies in *Populus* spp. revealed strong genetic associations between phenology and variants of several genes, including *PHYTOCHROME B2* (*PHYB2*), which regulates light input (Ingvarsson et al. 2008, Ma et al. 2010), *LHY1* and *LHY2*, both elements of the circadian clock (Ma et al. 2010), and *FLOWERING TIME 2* (*FT2*) (Wang et al. 2018). These relationships are likely to be evolutionarily important. Genome-wide association studies have uncovered natural variation in genes controlling growth in black cottonwood (*P. trichocarpa*) (Evans et al. 2014, McKown et al. 2014) and European aspen (*Populus tremula*) (Wang et al. 2018). In *P. trichocarpa*, variation in several circadian clock gene loci, including *PRR5*, *PRR7* and *LUX ARRHYTHMO/ PHYTO CLOCK 1* (*LUX/PCL1*), is associated with bud set and leaf drop; variation in *PRR7* is also associated with growing period and plant height (McKown et al. 2014). The *FT2* locus explains a high proportion of the genetic variation in timing of bud set in the *Populus tremula* (SwAsp) collection (Luquez et al. 2008) and one variant is particularly associated with populations originating in Northern Sweden, where growth is restricted to very short periods of very long daylengths (Wang et al. 2018).

### Tracking of photoperiod and timing of flowering and growth—the same but different?

Flowering is a well-characterized seasonally regulated event that in many plants is regulated by daylength, which provides an accurate indication of the time of year. Plants adapted to growth at high latitudes will flower during the warm days and long photoperiods of the spring and summer (Thomas and Vince Prue 1997, Imaizumi and Kay 2006). Species from lower latitudes are often less daylength-sensitive or may flower during short photoperiods. Increasing altitude has a similar effect on flowering to increasing latitude, as temperature, irradiance and water availability are all affected by height above sea level and plants must ensure their growth and reproduction are timed to occur at the most favourable time of year (Thomas and Vince Prue 1997). Even near the equator, some plant species may still show seasonal rhythms either because growth is restricted by climatic factors or because individuals within a population must synchronize flowering to ensure successful reproduction (Borchert et al. 2005). Across the globe, therefore, plants gauge both the quality of light and the duration of the photoperiod (Adole et al. 2019). This information is then used to reset the circadian oscillator to local time, ensuring all clock-controlled output pathways are coordinated with the external environment (Borchert et al. 2005).

**Figure 3.**
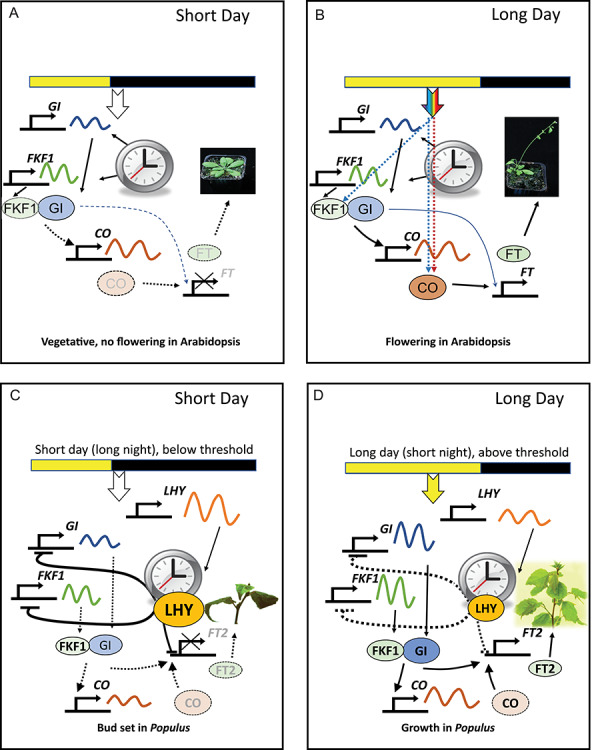
Photoperiodic control of flowering and growth. The pathways controlling (A, B) flowering time in Arabidopsis and (C, D) growth cessation in *Populus* spp. involve similar components. Short days promote (A) vegetative growth in Arabidopsis and (C) bud set in *Populus* spp*.*; long days promote (B) flowering in Arabidopsis and (D) bud break and growth in *Populus* spp. Photoperiodic responses in each species are controlled by external coincidence. In long days in Arabidopsis, FKF1 and GI stabilize CO, leading to FT expression which promotes flowering. In *Populus* spp, LHY2 controls *FT2* expression directly as well as via control of coincidence of FKF1 and GI. FT2 promotes growth. In SDs in Arabidopsis, CO degrades before it is able to promote FT. Further details are provided in the text. Arrows show positive effects; bars show negative effects. Black, dotted lines indicate inactivate pathways; blue and red dotted lines indicate light transduction via photoreceptors promoting protein stability; rainbow or yellow arrow indicates perceived light. Inactivated products appear in grey.

In the morning, light is enriched in the blue region of the spectrum, at midday in the blue-red region and in the evening in the far-red region. These light quality signals supplement the diverse metabolic cues entraining the clock (Oakenfull and Davis 2017). There are two main models describing the response of the circadian oscillator to light. The ‘external coincidence model’, which was initially proposed by Edward Bünning, states that the circadian oscillator sets the rhythm of a deciding (transcription) factor and determines a critical, light-sensitive phase of its cycle (Bünning 1936, Thomas and Vince Prue 1997, Davis 2002). Only when the factor is present during the light-sensitive phase in the presence of light of sufficient duration, irradiance and quality is a response induced. This model is complemented by the ‘internal coincidence model’ (Pittendrigh and Minis 1964). This postulates that the oscillator sets the rhythm of several clock-regulated (transcription) factors and that the physiological response is induced when specific phases of these internal rhythms coincide. Detailed genetic studies of floral induction in Arabidopsis indicate that the initial stage results from the co-expression of the circadian clock-controlled genes *FKF1* and *GIGANTEA* (*GI*). FKF1 and GI form a protein complex that activates expression of CO. CO is essential for flowering as it activates expression of FLOWERING LOCUS T (FT), which initiates flower formation at the apical meristem. CO is only available in sufficient quantity to activate FT when it is stabilized by far-red or blue wavelengths of light in the morning and at the end of the day (Suarez-Lopez et al. 2001, Valverde et al. 2004, Song et al. 2018). This matches the predictions of the external coincidence model: CO is stabilized at a point in the circadian cycle when it can induce expression of FT only under long-day photoperiods (Figure 3C).

Photoperiodic information is perceived by photoreceptors in the leaves, which are thus the most important and primary site of light reception. Clock resetting and clock-induced production of FT also occurs in the leaves. FT is transported via the phloem to the apical meristem where it modulates expression of the floral identity genes. FT associates with FD and together they initiate the switch of cell fate of the meristematic cells from indeterminate vegetative growth to the determinate flower formation (Abe et al. 2005, Wigge et al. 2005)

Although use of hybrid aspen as a model tree has enabled considerable progress to be made in identifying the components regulating the photoperiodic control of growth, the spatial perception of the daylength signal in regulating seasonal growth has only recently been adequately addressed. Two main pathways are involved in photoperiodic responses in hybrid aspen, the CO/FT and the gibberellin (GA) pathways (Böhlenius et al. 2006, Eriksson et al. 2015). Spatial control of photoperiodic perception and its contribution to the seasonal control of growth were considered in a recent study (Miskolczi et al. 2019). The authors exploited the differences in CDL between aspen ecotypes from the north, which respond early to SDs, and south of Sweden, which respond later to SDs, by grafting between plants with these contrasting phenotypes. The photoperiodic signals controlling seasonal growth were indeed perceived in the leaves. The question of how the leaf-mediated perception of the photoperiodic signal was transduced to the apex was resolved by demonstrating the mobility of FT. Importantly, the graft-transmissible movement of FT was essential for the transduction of photoperiodic signals from leaves to apex.

Despite these findings, FT may not be the exclusive long-range mobile signal; the same study showed that GA also acted systemically to control seasonal growth, although the long-range effects of FT were not mediated via GA acting as a secondary messenger from the leaves. Gibberellin was, however, a far less effective long-range signal than FT. It is thus plausible to suggest that FT is the predominant systemic signal from leaves to the apex. At the apex, the long-range FT signals positively regulate the GA pathway. This study has answered the long-standing question concerning the perception of seasonal cues and established the identities of the long-range signals transducing information from leaf to apex.

In perennial plants, seasonal and photoperiodic cues trigger a developmental switch from active vegetative growth to growth cessation. This results in inactivity of the apical meristems following exposure to photoperiods below the CDL. When the shoot apical meristem (SAM) arrests, any previously formed primordia continue to grow and form bud scales that surround the meristem and protect it during winter. The meristem is further insulated by callose depositions in the plasmodesmata of the surrounding cells. Callose deposition is controlled by abscisic acid (ABA) signalling and the deposits are removed when dormancy is broken (Singh et al. 2019).

In trees, as in Arabidopsis, both GAs and the FT-regulated module promote growth (Eriksson et al. 2015, Miskolczi et al. 2019). The GA biosynthesis-limiting enzyme GA 20-oxidase 1 (GA20ox1) in *Populus* spp*.* is regulated by daylength and, in *Salix* and *Populus* spp*.*, levels of bioactive GAs are down-regulated after a few SDs. This mechanism ensures a rapid cessation of growth and is a prerequisite for bud set and dormancy establishment, as trees over-expressing *Arabidopsis thaliana (At) AtGA20ox1* are unable to arrest growth even when the photoperiod is below the CDL (Eriksson et al. 2015).

Experimental analysis of the photoperiodic control of growth of trees reveals a similar mechanism to that controlling flowering in Arabidopsis, a facultative long-day plant. Studies of transgenic trees under-expressing clock-associated genes in artificial seasonal and daily cycles support this model of photoperiodical regulation. Photoreception by phyA is required for maintenance of active growth; phyA also affects expression of *LHY1* and *LHY2*, causing the circadian period (and therefore the phase) to change in response to light (Kozarewa et al. 2010). The circadian clock itself modulates the daylength requirement for growth, thus faster clocks have an earlier phase of *FKF1* and *GI*, which control expression of *CO* and *FT2* and, therefore, growth (Ibáñez et al. 2010, Kozarewa et al. 2010). The pattern of *CO* and *FT2* expression matches that predicted by the external coincidence model. FKF1 and GI directly regulate *FT2* (Böhlenius et al. 2006, Ding et al. 2018). Recently, an additional and novel role of a tree orthologue of Arabidopsis branching regulator (*BRC1*, a member of *TEOSINTE BRANCHED 1, CYCLOIDEA, PCF family*) has been shown in photoperiodic regulation of growth cessation in poplar trees (Cubas 2020, Maurya et al. 2020). BRC1 acts downstream of the *CO/FT* module and in a negative feedback loop interacts with FT protein to antagonize its action (Maurya et al. 2020). Photoperiodic pathways involving clock genes and their regulation of CO/FT involved in growth cessation in trees are shown in Figure 3B.

In *Populus tremula x P. alba* (INRA 717-184), LHY2 is necessary and sufficient to activate night length signalling to inhibit FT2 and the growth-promoting pathway following exposure to SDs (Ramos-Sánchez et al. 2019). All the data indicate that, in trees, the circadian clock components LHY1 and LHY2 directly control the expression of *GI*, *FKF1*, *CO* and *FT2* to determine growth. Recent studies have, however, revealed a novel pathway that involves regulation of *FT2* expression by FKF1 and GI (Ding et al. 2018) (Figure 3B). There are thus several layers of control regulating growth in response to changing photoperiods.

**Figure 4.**
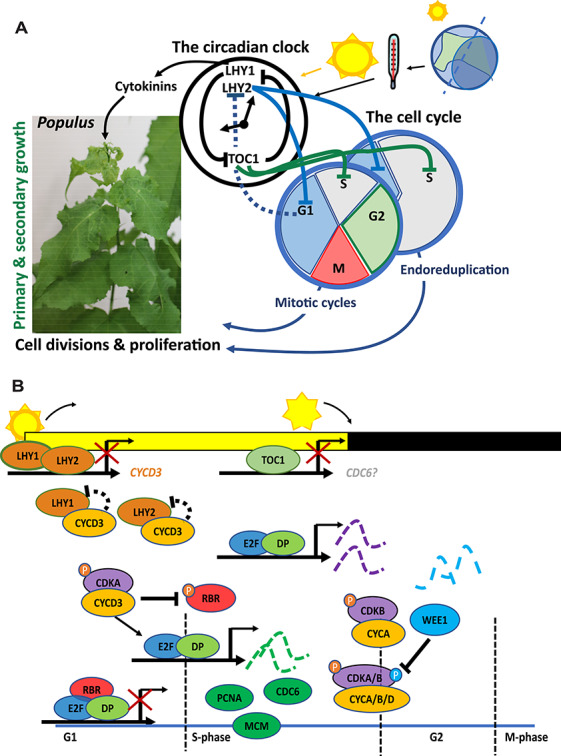
(A) The plant circadian clock affects primary and secondary growth by regulating the cell cycle. This figure summarizes how the core circadian clock may interact with the cell cycle to regulates cell proliferation and growth in *Populus* spp.; further details are provided in the text. (B) The morning-phased and evening-phased clock components regulate the cell cycle. The morning-phased components LHY1 and LHY2 in *Populus* spp. repress *CYCD3* expression, thus restricting CYCD3 to the early day. During the G1 phase, CYCD3 interacts with cyclin-dependent kinases (CDKs) to phosphorylate RETINOBLASTOMA RELATED 1 (RBR1), targeting it for degradation by the proteasome. The subsequent release from repression of replication factors in the heterodimer of the E2F–DP complex promotes expression of proliferating cellular nuclear antigen, mini-chromosome maintenance protein complex and CDC6, enabling their build-up prior to DNA replication in the S phase. TOC1 is an evening-phased component that may represses *CDC6* (in grey, with question mark) transcription, restricting CDC6 production to the daytime. Several replication factors together with CDC6 act to enable cells to undergo DNA replication in the S phase. The E2F–DP complex also promotes expression of CDKs, which act with CYCA/B/D to control the transition between the S and G2 (G2 to M) phases; the kinase WEE1 may phosphorylate CDKAs at this stage. Note: (B) is adapted from Dante et al. (2014).

In *Populus* spp*.*, the circadian clock controls key plant hormones affecting growth. Trees with shorter circadian periods and earlier phases of gene expression grow poorly in growth-promoting long days (Edwards et al. 2018). More detailed studies showed that the trees with reduced growth rates were deficient in active cytokinins, had an altered profile of *CYCD3* expression in leaves and the apical part of stems, and showed changes to the pattern of cell division in the cambium. LHY2 directly interacts with CYCD3, which acts during mitotic and endoreplication cycles to increase the cell division rate and thus growth. It is likely that the circadian clock acts via LHY1 and LHY2 to affect cytokinin biosynthesis directly. The clock also appears to regulate the cell cycle by controlling *CYCD3* expression, thus influencing cell proliferation and biomass production (Figure 4). It remains to be determined if this mechanism relies on external coincidence and, if so, which proteins are involved.

CYCD3 regulates the G1 to S phase of the cell cycle. In *Populus* spp*.*, as noted above, CYCD3 is controlled by LHY1 and LHY2, two MYB transcription factors that are key clock components*.* The situation appears similar in Arabidopsis, as all three *AtCYCD3* genes have CCA1-binding sites in their promoters. That these genes are targets of CCA1 and LHY has been confirmed experimentally in Arabidopsis: CCA1 binds the *CYCD3;3* promoter (Kamioka et al. 2016) and LHY binds to the promoters of *CYCD3;2* and *CYCD3;3* (Adams et al. 2018). Both *CYCD3;1* and *CYCD3;2* show rhythmic expression in Arabidopsis, and these rhythms are altered in plants over-expressing *TOC1* (Fung-Uceda et al. 2018). Moreover, TOC1 binds to the promoter of cell division cycle *6* (*CDC6*), a gene encoding a replication protein, to modulate cell proliferation and growth in Arabidopsis leaves (Fung-Uceda et al. 2018). Detailed studies of endoreduplication of leaf cells of different Arabidopsis clock mutants support this novel role for TOC1. These data reinforce the conclusion from *Populus* spp*.* that the circadian clock in plants directly regulates primary and secondary growth by controlling DNA replication and the cell cycle, thus determining mitotic events and endoreduplication at the meristem, and the growth of somatic cells (Edwards et al. 2018, Fung-Uceda et al. 2018). Recent studies also suggest an interaction between clock proteins and the cell cycle occurs during the differentiation of stem cells into vascular cells (Torii et al. 2019). The action of the circadian clock also safeguards the integrity of DNA, DNA replication and, ultimately, cell cycle progression by gating responses to UVB light (Fehér et al. 2011, Takeuchi et al. 2014). Figure 4 shows the pathways whereby the circadian clock may control the cell cycle and growth in trees, similar to and extending the findings in Arabidopsis.

**Figure 5.**
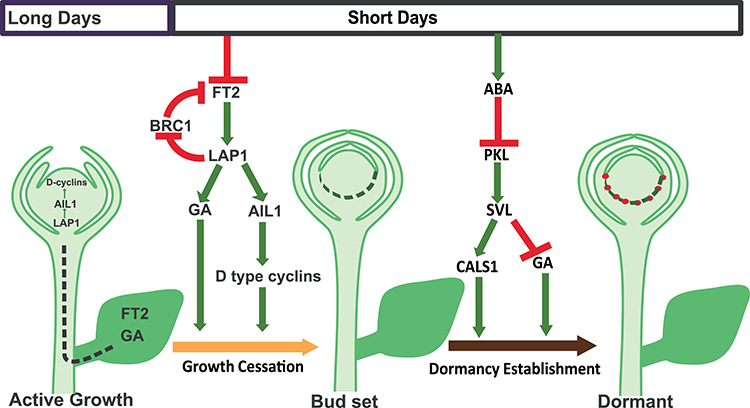
Regulation of active growth, growth cessation and dormancy in apical buds of hybrid aspen. During long days, FT and GA are produced in leaves and transported to the apex where they activate the LAP1–AIL1 pathway. This regulates active growth by controlling genes involved in the cell cycle. Short days lead to reductions in FT and GA levels in leaves. This blocks the active growth pathway (LAP1–AIL1 pathway), resulting in growth cessation and bud set. Additionally, reduction of LAP1 leads to upregulation of BRC1, which in turn interact with FT protein and antagonize its action, providing an additional control for seasonal growth. A subsequent increase in ABA level suppresses PKL and induces SVL, which establishes dormancy. During dormancy establishment, the plasmodesmata are closed by callose deposition by CALS1 and GA catabolism genes are activated to degrade any active GA remaining in the apex. The pathways shown are based on studies of hybrid aspen but most of the components are known to regulate similar responses in other species of tree.

*AINTEGUMENTA LIKE 1* (*AIL1*) is expressed under growth-promoting conditions but down-regulated under SDs (Karlberg et al. 2011). Trees over-expressing *PHYA* or *FT2* under control of the constitutive 35S CaMV promoter have normal levels of *AIL1*, and *FT2* is regulated normally in trees over-expressing *AIL1*, suggesting AIL1 acts downstream of light perception and the CO/FT module. Reducing *AIL1* transcript levels by RNA interference (RNAi) results in decreased *CYCD3* expression, which is associated with reduced cell division; over-expression of *AIL1* has the opposite effects (Karlberg et al. 2011). AIL1 must therefore promote vegetative growth in a daylength-dependent fashion. AIL1 binds to the *CYCD3* promoter resulting in *CYCD3* expression, providing a mechanism for its growth-promoting role (Figure 5). In hybrid aspen, *LIKE-AP1* (*LAP1*), an orthologue of the Arabidopsis floral meristem identity gene *APETALA1* (*AP1*), acts downstream of the CO/FT to modulate growth in a photoperiod-dependent manner (Azeez et al. 2014). LAP1 acts upstream of AIL1, however, as trees with low levels of *LAP1* due to RNAi show early growth cessation, but over-expression of *LAP1* leads to continuous growth (Figure 5). Levels of *LAP1* are normal in trees over-expressing *PHYA* or *FT2*. Moreover, LAP1 appears to regulate *AIL1*, given that *AIL1* expression was not down-regulated in trees over-expressing *LAP1* but reduced *LAP1* expression resulted in lower levels of *AIL1* during SDs. Additionally, LAP1 has also recently been shown to be involved in regulation of BRC1, which can interact and antagonize FT protein. Under long photoperiod and growth-promoting conditions, LAP1 can directly bind to BRC1 promoter to suppress its expression (Maurya et al. 2020). Short photoperiod results in downregulation of FT and hence LAP1 expression. This reduction in LAP1 removes suppression of BRC1, which is turn gets upregulated and then interacts with FT protein and antagonize its action (Figure 5). Direct regulation of the cell cycle and growth by the plant circadian clock enables a tight coupling to the daily and yearly environmental cycles (Edwards et al. 2018); this is important for all plants but perhaps particularly so for tree species whose lifespans may extend over hundreds—or indeed thousands—of years.

### Regulation of growth by phytohormones

The importance of plant hormones as internal modulators of plant growth and development cannot be overstated. The underlying networks were initially identified in the model plant Arabidopsis. The picture is continually updated by new studies that develop our understanding of plant plasticity in terms of hormonal effects on growth and development or responses to variation in the environment. Modulation of growth depends on the proper control of growth regulators. Auxin, cytokinins (CKs) and GA are key plant hormones that promote both primary and secondary growth; in contrast, ABA is an important limiter of growth. Although other plant hormones also play important roles, this review will focus on these four main hormones.

The plant circadian clock plays an important role in the production of, and the sensitivity and responses to, most hormones (Singh and Mas 2018). Gibberellins act in parallel to the *CO/FT* module to control timing of vegetative growth and flowering, as described above. Gibberellins act generally to promote growth, flowering and fruit development, and xylem fibre development in both Arabidopsis and perennial plants (Ridoutt et al. 1996, Eriksson et al. 2000, Hedden and Sponsel 2015, Felipo-Benavent et al. 2018). Although the circadian clock gates sensitivity to GA, rendering plants more GA-sensitive at night, GA appears not to affect the circadian oscillator directly (Hanano et al. 2006, Arana et al. 2011).

Auxins are involved in cell elongation, cell division and xylem fibre development, and also contribute to growth regulation. These activities are enabled in part by circadian modulation of auxin sensitivity (Covington and Harmer 2007, Nozue et al. 2007). Sensitivity to auxins is ‘turned off’ in *Populus* spp. during winter to help maintain dormancy (Baba et al. 2011). Cytokinins act together with auxins to regulate cell division and growth (Bhalerao and Fischer 2014, Randall et al. 2015). They also affect the circadian clock (Hanano et al. 2006, Zheng et al. 2006), and, in *Populus* spp*.*, their biosynthesis apparently depends on a functional clock and expression of *LHY1* and *LHY2* in the morning (Edwards et al. 2018). Reductions in bioactive CK levels reduce the rate of cell division and lead to lower biomass accumulation (Nieminen et al. 2008). Together, these findings illustrate the significant contributions of the circadian clock to successful growth and reproduction by complex regulatory pathways.

## Bud development

Proper bud development, including bud set and dormancy establishment, is essential for tree survival during adverse conditions. Exposure to short photoperiods initiates the growth cessation process, during which the leaf primordia at the apical meristem undergo a transition to form bud scales instead of leaves, leading to bud set. In trees from temperate regions, the bud scales are usually hairy with thick cuticles to provide the bud with extra protection from extreme cold. Overall this process is termed ‘bud set’. Subsequent to setting, buds are able to withstand adverse conditions such as low temperature by maintaining dormancy (Rohde et al. 2000, Rohde and Bhalerao 2007). The bud itself may be vegetative or reproductive. Although it is often very difficult to distinguish bud type prior to flush, small differences in morphology may be apparent: flower/reproductive buds are fatter and rounder than the thinner, more pointed vegetative buds.

### Role of light and temperatures in bud development

Bud formation involves bud set, acclimation to dehydration and cold, and the establishment of dormancy. Bud set begins immediately after the detection of short-day photoperiods, whereas dormancy establishment occurs a few weeks later (Ruttink et al. 2007). Metabolic and gene expression studies of these different levels of bud formation have shown that each stage involves distinct signalling and activation pathways. Bud set mainly involves suppression of the genes responsible for maintaining the stem cell population in the central meristem (Figure 5); these genes include *CYCD3*, which is regulated by *AIL* genes in the apex (Karlberg et al. 2011). *AIL1* is regulated by the photoperiodic pathway downstream of *FT* and *LAP1*, indicating that daylength is important in controlling the cell cycle during bud set (Azeez et al. 2014). Temperature is also a regulatory factor in growth cessation and bud set in fruit trees such as apple and pear (*Pyrus communis*) (Heide and Prestrud 2005). Photoperiod and temperature may affect bud set independently or in combination. A study in which 52 clonally replicated poplar genotypes were grown in different latitudes found that temperature influences the timing of bud set by modifying the CDL (Rohde et al. 2011).

Dormancy establishment is an independent process that occurs after bud set. Both ABA-insensitive *abi1-1* mutants and *SVL-RNAi* plants (in which *SHORT VEGETATIVE PHASE-LIKE (SVL)* was down-regulated by RNAi) show defects in dormancy establishment but no changes in bud set or growth cessation responses (Tylewicz et al. 2018, Singh et al. 2019). Over-expression of the *SVP* homologues *MdSVPa* and *MdSVPb* in kiwi (*Actinidia deliciosa*) and apple affected dormancy and bud break without changing growth cessation and bud set (Wu et al. 2017*a*, 2017*b*). Plants over-expressing the dominant negative form of *ABSCISIC ACID INSENSITIVE 3* (*ABI3*) or *ETHYLENE RECEPTOR 1* (*ETR1*), however, show aberrant bud set but no changes to bud dormancy (Rohde et al. 2002, Ruttink et al. 2007).

The importance of photoperiod and temperature in dormancy establishment varies across species; short photoperiods are sufficient to induce and establish dormancy in poplar (*Populus* spp*.*) and spruce (*Picea* sp.) (Heide 1974, Espinosa-Ruiz et al. 2004), but temperature plays the key role in the Rosaceae (Heide and Prestrud 2005). Additional insight in the photoperiodic control of dormancy establishment has been provided recently by establishing the role played by ABA and its downstream partners (Tylewicz et al. 2018, Singh et al. 2019); in contrast, the role of temperature in dormancy establishment is still not properly understood. A study conducted under controlled environmental conditions indicated that moderate temperatures (~18 °C) are best for inducing bud set and dormancy establishment (Rinne et al. 2018). Low temperatures during bud set affect bud quality and dormancy establishment, whereas higher temperatures during bud set result in bud flush (Rinne et al. 2018). Dormancy release components are active if the buds are subjected to lower temperatures before the complete establishment of dormancy. Such results suggest that trees adapt to local conditions in order to set bud and establish dormancy before the temperature goes down to chilling levels; this is consistent with other studies (Tylewicz et al. 2018, Singh et al. 2019). During the establishment of dormancy, it is necessary for trees not only to stop producing factors promoting growth but also to maintain a second level of defence by isolating the SAM from growth-promoting signals. Pathways induced either by low temperature stress or by warm temperatures but short photoperiods act redundantly to ensure responses are sufficiently plastic to enable adaptation to local conditions (Tanino et al. 2010). Exposure to SDs and warm night-time temperatures strongly accelerates growth cessation, dormancy development and cold hardiness in hybrid poplar. In contrast, a combination of long daylengths with low night-time temperatures bypasses the short photoperiod requirement completely in northern ecotypes of dogwood, but not in southern ecotypes. This suggests genetic differences in responses to temperature and photoperiod are important to adaptation to local environments. Although it is clear that temperature plays a role in dormancy establishment, the mechanism remains unclear. Whether it resembles the SD- induced ABA pathway or involves different regulatory elements remains to be studied.

### Role of phytohormones in bud development

Phytohormones play a crucial role in bud set and dormancy establishment. Abscisic acid and GA act antagonistically during these processes as GA promotes growth but ABA induces dormancy. Short photoperiods and low temperatures activate the ABA signalling and biosynthesis pathways that induce bud dormancy in many plant species, including poplar, kiwi, grapes and apple (Rohde et al. 2002, Rohde and Bhalerao 2007, Ruttink et al. 2007, Li et al. 2009, Singh et al. 2017, 2018, Tuan et al. 2017, Wang et al. 2017, Rehman et al. 2018, Tylewicz et al. 2018). Levels of ABA are high during the induction and development of dormancy but decrease during dormancy release and bud break (Or et al. 2000, Zheng et al. 2015, Wang et al. 2016, Wen et al. 2016, Chmielewski 2017, Li et al. 2018, Zhang et al. 2018). Analyses of the transcriptome and expression of individual genes during dormancy induction in several plant species show upregulation of genes involved in ABA biosynthesis (NCEDs), signalling and reception (RCARs) (Bai et al. 2013, Zhong et al. 2013, Zheng et al. 2015, Wang et al. 2016, Vergara et al. 2017, Wu et al. 2017*a*, Li et al. 2018, Rehman et al. 2018, Zhang et al. 2018). Abscisic acid acts by blocking cell–cell communication during the establishment of bud dormancy in poplar (Tylewicz et al. 2018), and ABA-insensitive mutants with defects in dormancy establishment were unable to close the plasmodesmata around the SAM, providing solid evidence for the involvement of ABA in this process. Moreover, down-regulation of *SVL* impairs dormancy in poplar and *SVL* is activated via ABA during dormancy establishment (Singh et al. 2019). Recent findings in other species further confirm the role of ABA in dormancy establishment and a conserved and central role for ABA in seeds and buds.

Gibberellins are growth-promoting phytohormones whose bioactive levels decrease during growth cessation and dormancy establishment. Down-regulation of GA pathways occurs early in growth cessation and largely depends on photoperiod in poplar (Zawaski and Busov 2014, Eriksson et al. 2015). The transcriptional dynamics of GA biosynthesis and catabolism genes during dormancy establishment suggest overall decreases in *GA20 OXIDASE * and *GA3 OXIDASE* but an increase in *GA2 OXIDASE* (Bai et al. 2013, Zhong et al. 2013, Zheng et al. 2015, 2018, Zhu et al. 2015, Wen et al. 2016, Rehman et al. 2018, Singh et al. 2018, Zhang et al. 2018). Although GA levels decrease during growth cessation, which occurs significantly earlier than dormancy establishment, plants must prevent upregulation of bioactive GA during dormancy establishment. Recently, it was shown that GA, like FT, systemically controls growth cessation and bud break in poplar (Singh et al. 2018, Miskolczi et al. 2019), therefore GA biosynthesis and levels of bioactive GA are tightly controlled during dormancy establishment. A recent study in poplar revealed this control is achieved via direct upregulation of *GA2 OXIDASE* expression by SVL, which was previously known to regulate photoperiodic dormancy (Singh et al. 2018).

Although the data suggest the more robust role in dormancy establishment is played by ABA, with the more minor involvement of GA, it is possible that the change from growth to dormancy relies not on the individual levels of each hormone but on their relative ratio (Shu et al. 2013, Khalil-Ur-Rehman et al. 2017, Vimont et al. 2018). The hormonal balance between ABA and GA is regulated by endogenous and environmental signals. The same factor(s) may regulate both hormones antagonistically; for example, during dormancy establishment and bud break in poplar, SVL directly regulates expression of *NINE-CIS-EPOXYCAROTENOID DIOXYGENASE* 3 (*NCED3*), which encodes a protein involved in ABA biosynthesis, and *GA2 OXIDASE8*, which is involved in GA catabolism (Singh et al. 2018, 2019). More detailed studies involving metabolomics, transcriptomics and genetic analysis are required for a full understanding of the regulatory processes involved.

### Epigenetic mechanisms controlling bud dormancy establishment

Epigenetic regulation of dormancy establishment in trees has recently received significant attention. Early studies showed that histone modifications, DNA methylation and chromatin remodelling were involved in controlling transcription of the genes involved regulating this process. Higher levels of gDNA methylation occur during dormancy establishment in chestnut buds than in actively growing ones (Santamaria et al. 2009). Similarly, in poplar, higher levels of methylation and lower levels of H4K8Ac are observed in stems during winter dormancy than during active growth (Conde et al. 2013, 2017*b*, Kumar et al. 2016). Consistent with these observations, over-expression of *C. sativa*  *DEMETER-LIKE 10* (*CsDML10*), a DNA demethylase, leads to early apical bud maturation in poplar. This does not affect the overall DNA methylation changes induced by SD, thus suggesting a locus-specific activity for this protein during bud development (Conde et al. 2017*b*).

Components of the *POLYCOMB REPRESSIVE COMPLEX 2 (PRC2)*, such as *FERTILIZATION-INDEPENDENT ENDOSPERM (FIE)*, are upregulated during growth cessation and dormancy establishment. Moreover, use of RNAi to suppress *FIE* expression alters dormancy establishment in hybrid aspen buds but does not affect growth cessation and bud formation (Petterle 2011). Other chromatin-modifying genes, including *histone deacetylase* 14 (*HDA14*), *HDA08*, *HISTONE-LYSINE N-METHYLTRANSFERASE* (*SUVR3*) and *HISTONE MONO-UBIQUITINATION 2* (*HUB2*), are also up-regulated during the transition to dormancy in *Populus* (Ruttink et al. 2007, Karlberg et al. 2010). Expression of the chromatin remodelling gene pickle (*PKL*), an antagonist of *PRC2*, is down-regulated in short photoperiods in wild-type plants but upregulated in *abi1-1* mutants, which show dormancy defects. Down-regulation of *PKL* expression restores short photoperiod-induced dormancy in *abi1-1* mutants, suggesting that ABA promotes dormancy by repressing *PKL* (Tylewicz et al. 2018).

## Molecular regulation of dormancy establishment

Dormancy establishment occurs in response to photoperiod, temperature, phytohormones and epigenetics, as outlined above. These factors interact to modify the expression of a suite of molecular factors which direct the process that establishes dormancy. The molecular factors regulating dormancy have been identified recently in different tree species (Shim et al. 2014). A region consisting of six tandemly arrayed genes containing MADS- box domains, known as the *DORMANCY ASSOCIATED MADS BOX* (*DAM*), is partially deleted in the peach (*Prunus persica*) *evergreen* (*evg*) mutant (Bielenberg et al. 2006). Putative orthologues of these genes have been identified and studied with respect to dormancy establishment in a variety of plants, including leafy spurge (*Euphorbia esula*) (Horvath et al. 2010), Japanese apricot (*Prunus mume*) (Yamane et al. 2008, 2011, Sasaki et al. 2011), pear (Niu et al. 2016), apple and kiwi (Wu et al. 2012, 2017*a*, 2017*b*). The genes making up the *DAM* are closely related to the Arabidopsis floral regulators *SVP* and *AGAMOUS-LIKE 24* (*AGL24*). Several *SVL/SVP* genes play a role in establishing or maintaining dormancy. In poplar, SVL acts downstream of ABA to regulate bud dormancy induced by short photoperiods by directly regulating the expression of *GA2 OXIDASE*, a GA catabolism gene, and *CALLOSE SYNTHASE 1* (*CALS1*) (Singh et al. 2018). Similarly, in plants such as apple and kiwi, expression levels of *SVP* correlate with dormancy establishment and the fact that its over-expression results in delayed bud break suggests it has a role in regulating bud dormancy (Wu et al. 2017*a*). The roles played by *DAM* and *SVL* in temperate fruit trees have recently been discussed in detail elsewhere (Falavigna et al. 2019). Expression of several other genes is also modified during dormancy establishment. Genes involved in ABA and GA metabolism are differentially expressed (Singh et al. 2018, Khalil-Ur-Rehman et al. 2019), as are genes involved in plasmodesmata closure, including *CALS1* and *GLUCANASES*; these changes are discussed above. The same genes are differentially expressed in poplar plants such as *abi1-1* and *SVL-RNA*i that have dormancy defects (Singh et al. 2018, Tylewicz et al. 2018). At present, the function of these genes is still only assumed from correlation studies and functional validation is required to establish their exact roles in controlling dormancy. Despite this ‘caveat’, a model representing the various molecular factors likely to be involved in bud formation, bud set and dormancy establishment in hybrid aspen has been proposed (Figure 5). Bud set (growth cessation) and dormancy establishment are independent processes occurring in response to photoperiodic changes. During the first phase, a decrease in photoperiod reduces expression of FT and GA expression, which leads to suppression of growth via pathways involving LAP1, AIL1 and CYCD3. Additionally, BRC1, which has been reported recently, is involved in photoperiodic regulation of FT protein in apex and is a part of negative feedback loop involving LAP1. In the second phase, an increase in ABA levels suppresses PKL and thus induces expression of *SVL*, which causes a decrease in GA levels and promotes CALS1 activity in the buds leading to dormancy.

### Role of temperature and photoperiod in dormancy release and bud break

Release from a dormancy requires a certain period of chilling temperatures. The optimum temperature and duration of chilling required to end dormancy varies between tree species but non-freezing temperatures between 0 and 8 °C will release most trees from dormancy (Saure 1985, Espinosa-Ruiz et al. 2004, Brunner et al. 2014, Fu et al. 2015). For some species, including birch (*Betula*) and white spruce (*Picea glauca*), exposure to the same temperature that establishes dormancy induces dormancy release and reactivates growth (Heide 1993, Myking and Heide 1995, Cooke et al. 2012). How temperature is sensed during dormancy release and bud break remains unknown. As the process requires exposure to an extended period of low temperature to induce release from dormancy and to a similar period of warmer temperatures to induce bud break, the regulatory mechanism it is not easy to determine experimentally. Low temperatures induce release from dormancy via opening of the plasmodesmata, which were closed by callose deposition during dormancy establishment (Rinne et al. 2001, Singh et al. 2018, Tylewicz et al. 2018). The plasmodesmatal opening restores the responsiveness of the SAM to growth-promoting signals (Rinne et al. 2011). In most plant species, temperature rather than photoperiod controls dormancy release. In some species, however, long-day photoperiods also play a role in growth reactivation via an unknown mechanism (Saure 1985). Although light is mostly not required for dormancy release, it is important afterwards during bud break.

### Role of phytohormones in dormancy release and bud break

Abscisic acid and GA work antagonistically to regulate bud break. Exogenous application of GA leads to early release from dormancy and bud break, whereas application of ABA delays bud break (Rinne et al. 1994*a*, 1994*b*, 2011). Different forms of bioactive GA have different functions; in *Populus*, GA3 is involved in dormancy release and GA4 promotes bud break (Rinne et al. 2011). Application of GA3 enhances bursting of dormant Elberta peach buds and GA4 application enhances dormancy release of Japanese apricot flower buds (Zhuang et al. 2015). In kiwi, GA3 application after exposure to chilling also promotes bud break (Lionakis and Schwabe 1984). Studies of the role of ABA in bud break have produced conflicting results, which makes its effects more difficult to understand. Application of exogenous ABA delays bud break in birch (Rinne et al*.* 1994), apple (Dutcher and Powell 1972), kiwi (Lionakis and Schwabe 1984) and sour cherry (*Prunus cerasus*) (Mielke and Dennis 1978). A decrease in ABA levels preceding release from dormancy and bud break occurs in many plant species, including birch (Rinne et al. 1994*a*), grape vine (*Vitis vinifera*) (Koussa et al. 1994, Or et al. 2000, Destefano-Beltran et al. 2006, Li et al. 2018) and potato (Destefano-Beltran et al. 2006); in contrast, exogenous application of ABA to grape vines during spring has little effect on bud break (Hellman et al. 2006). Similarly, there is no clear effect of chilling on ABA levels in birch buds (Rinne et al. 1994*b*) despite chilling being a prerequisite for bud break. Although GA and ABA are thought to act antagonistically in bud break, whether this depends on the individual levels of each hormone or on the relative ratio of their expression remains to be determined.

### Epigenetic mechanisms controlling dormancy release and bud break

Chromatin remodelling is also implicated in dormancy release and bud break in a manner resembling the process of vernalization in Arabidopsis, during which *FLOWERING LOCUS C* (*FLC*) is repressed by trimethylation of histone (Michaels and Amasino 1999, Gendall et al. 2001, Bastow et al. 2004). An increase in H3K27me3 at particular loci in *DAM6* occurs at the time of dormancy release and bud break (Leida et al. 2012). Decreases in the trimethylation of lysine 4 in histone H3 (H3K4me3) have been found in the chromatin of *DAM1* in leafy spurge (Horvath et al. 2010), *DAM6* in peach (Leida et al. 2012), and *MADS13-1* in Asian pear (*Pyrus pyrifolia*; *PpMADS13-1*) (Saito et al. 2015). Up-regulation of *EARLY BUD-BREAK* (*PpEBB*), an AP2/ERF transcription factor, in Asian pear during floral bud break results from increased levels of active trimethylation of the histone H3 tail at Lys4 (H3K4me3) upstream of start codon region (Tuan et al. 2017).

Levels of gDNA methylation are reduced during reactivation of growth in the apical shoot during bud break in spring (Conde et al. 2017*a*). In poplar, low temperatures induce expression of *PtaDML10*; a functional analysis suggested that PtaDML10 primarily mediates bud break by reactivating transcription of key genes controlling protein homeostasis, meristem activity, and blue light- and L1-specific cell signalling (Conde et al. 2017*b*). In apple, DNA methylation levels decrease gradually between flower bud dormancy and fruit set (Kumar et al. 2016). A recent perspective providing insight into mechanisms by which chromatin modification regulates bud break by chromatin modification is worth reading (Conde et al. 2019).

### Molecular regulation of dormancy release and bud break

Many transcriptional and molecular changes occur during dormancy release and bud break. Low temperature alters the expression of genes involved in these processes (Rinne et al. 2001). As plasmodesmata are closed during the establishment of dormancy, their re-opening is essential for the resumption of the symplastic connections that enable entry of growth-promoting signals to SAM cells (Rinne et al. 2001, 2011, Singh et al. 2018, Tylewicz et al. 2018). Low temperature and GA induce expression of β-1-3 glucanases (GH_17) (Rinne et al. 2011, Singh et al. 2018), which promote dormancy release and bud break. Given that low temperature also influences GA metabolism by up-regulating expression of *GA20 OXIDASE*, it is uncertain whether low temperature regulates GH_17 expression directly or indirectly by the GA pathways (Rinne et al. 2011). Like *GA20 OXIDASE*, *FT1* expression is upregulated by low temperatures and promotes growth (Böhlenius et al. 2006, Rinne et al. 2011, Azeez et al. 2014, Eriksson et al. 2015, Miskolczi et al. 2019); thus both *GA20 OXIDASE* and *FT1* are likely to be involved in dormancy release and bud break.

A transcriptional network that may act in hybrid aspen and other trees to regulate dormancy establishment and bud break was identified recently by transcriptional and genetic analyses of known and novel molecular factors mediating dormancy release and bud break (Singh et al. 2018). The main component of this network is SVL, which acts as a central regulator of both growth-promoting and growth-suppressing genes. Low temperature suppresses *SVL* expression and induces *FT1* and GA biosynthesis and, by their action, bud break. SVL, however, can also suppress bud break by inducing expression of *TCP*  *DOMAIN PROTEIN 18 (TCP18)/ BRANCHED 1* (*BRC1*) (Singh et al. 2018) as well as maintain dormancy by positively regulating ABA biosynthesis and signalling. Over-expression of *PYRABACTIN RESISTANCE 1/PYR1-LIKE/REGULATORY COMPONENTS OF ABA RECEPTORS* (*PYR1/PYL/RCARs*) in poplar also delays bud break, suggesting a negative role for ABA in this process. *TCP18* regulates auxiliary branching in Arabidopsis and other plant species but was also identified as a novel molecular factor regulating temperature-mediated bud break in poplar.

**Figure 6.**
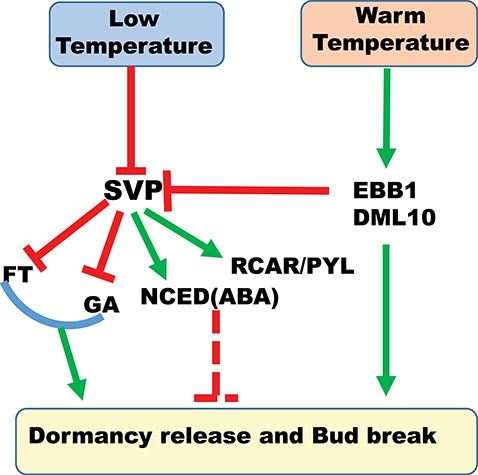
The molecular factors regulating the thermal control of bud break in hybrid aspen. Dormancy release results from exposure to extended low temperatures; warm temperatures promote bud break and growth. Low temperature suppresses expression of SVL, a negative regulator of the growth-promoting FT/GA pathway. SVL promotes ABA biosynthesis and receptors via a positive feedback loop resulting in high levels of ABA to maintain dormancy and inhibit dormancy release. The pathways shown are based on studies of poplar trees but most of the components are known to be actively involved in regulating similar responses in other species.

Several genes in addition to those acting in the networks described above have been implicated in dormancy release and bud break. In poplar, these include *CENTRORADIALIS 1* (*CEN1*), *LHY* and *EARLY BUD BREAK 1* (*EBB1*) (Ibáñez et al. 2010, Mohamed et al. 2010, Yordanov et al. 2014). Changes to the level of *PttCEN1* expression produce trees with different chilling requirements for dormancy release; trees over-expressing *PttCEN1* require a longer period of chilling than wild type, while RNAi plants require a shorter period. *PttCEN1* may be a negative regulator of bud break as its over-expression delays bud break in trees (Mohamed et al. 2010). Down-regulation of *PttLHY1* and *PttLHY2* delays bud break, but the mechanism of action is unknown (Ibáñez et al. 2010). *PttEBB1*, a poplar gene orthologous to *PpEBB* in Asian pear, is an ERF family member that was identified by activation tagging of mutant trees showing early bud break (Yordanov et al. 2014). Over-expression and down-regulation of *PttEBB1* in poplars results in early and late bud break, respectively. Changes to *PttEBB1* levels affect the expression of several genes associated with various metabolic processes, meristem growth and regulation of hormone levels. Some of the *DAM* genes, which induce dormancy, are down-regulated in trees over-expressing *PttEBB1*, suggesting *EBB1* induces bud break by suppressing these genes (Yordanov et al. 2014). Expression of *SVL*, which regulates bud break, was severely attenuated in trees with increases or reductions in *PttEBB1*, suggesting SVL acts downstream of EBB1. The actions of the various known and predicted molecular components involved in regulating bud break are summarized in Figure 6.

## Concluding remarks

The timing of growth in perennial plants and forest trees is directly related to productivity. Broad latitudinal clines in responses to seasonal variation in photoperiod and temperature have been revealed. These findings have implications for the maintenance of forest ecosystems and the ability of natural populations to adapt to climate change. Although many of the mechanisms underlying the ability of trees to match their growth to their environment are now understood, important questions still remain. Future investigations will provide important insight on the strategies used by plants to not only survive but also thrive in harsh environments.
